# Understanding the structural diversity of chitins as a versatile biomaterial

**DOI:** 10.1098/rsta.2020.0331

**Published:** 2021-09-20

**Authors:** Jiaxin Hou, Berk Emre Aydemir, Ahu Gümrah Dumanli

**Affiliations:** ^1^ Department of Materials, University of Manchester, Oxford Road, Manchester M13 9PL, UK; ^2^ Henry Royce Institute, University of Manchester, Oxford Road, Manchester M13 9PL, UK

**Keywords:** chitin, biomaterial, bioinspired optical, mechanical, crystalline properties

## Abstract

Chitin is one of the most abundant biopolymers, and it has adopted many different structural conformations using a combination of different natural processes like biopolymerization, crystallization and non-equilibrium self-assembly. This leads to a number of striking physical effects like complex light scattering and polarization as well as unique mechanical properties. In doing so, chitin uses a fine balance between the highly ordered chain conformations in the nanofibrils and random disordered structures. In this opinion piece, we discuss the structural hierarchy of chitin, its crystalline states and the natural biosynthesis processes to create such specific structures and diversity. Among the examples we explored, the unified question arises from the generation of completely different bioarchitectures like the Christmas tree-like nanostructures, gyroids or helicoidal geometries using similar dynamic non-equilibrium growth processes. Understanding the *in vivo* development of such structures from gene expressions, enzymatic activities as well as the chemical matrix employed in different stages of the biosynthesis will allow us to shift the material design paradigms. Certainly, the complexity of the biology requires a collaborative and multi-disciplinary research effort. For the future's advanced technologies, using chitin will ultimately drive many innovations and alternatives using biomimicry in materials science.

This article is part of the theme issue ‘Bio-derived and bioinspired sustainable advanced materials for emerging technologies (part 1)'.

## 1. Introduction

Chitin is the second most abundant biopolymer on earth second to cellulose, produced by crustaceans, molluscs, insects and some fungi [1]. In this opinion piece, we explore the structural diversity of chitin seen in nature and present the potential of it as an advanced material for various applications reflecting the structural and functional diversity.

**Figure 1.**
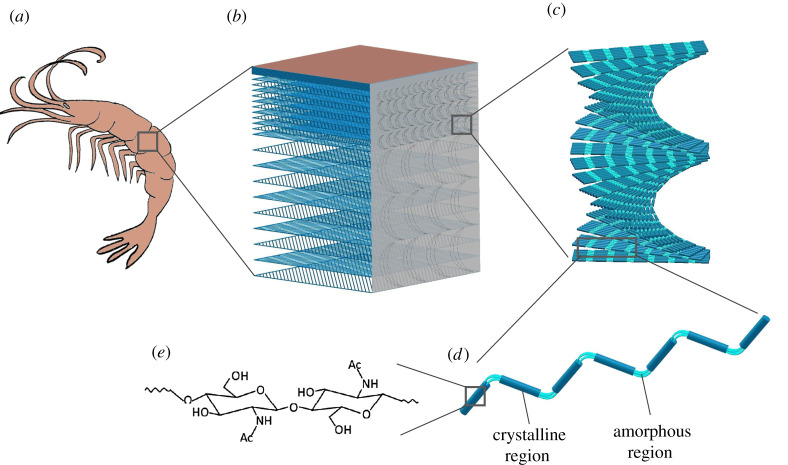
Hierarchical structure formation of chitin as a biocomposite on the (*a*) artistic illustration of a prawn exoskeleton; (*b*) the macrostructure of the exoskeleton primarily composed of layered chitin, proteins and CaCO_3_; (*c*) helicoidal arrangements of chitin; (*d*) chitin nanofibrillar bundles; and (*e*) chemical composition of chitin. (Online version in colour.)

**Figure 2.**
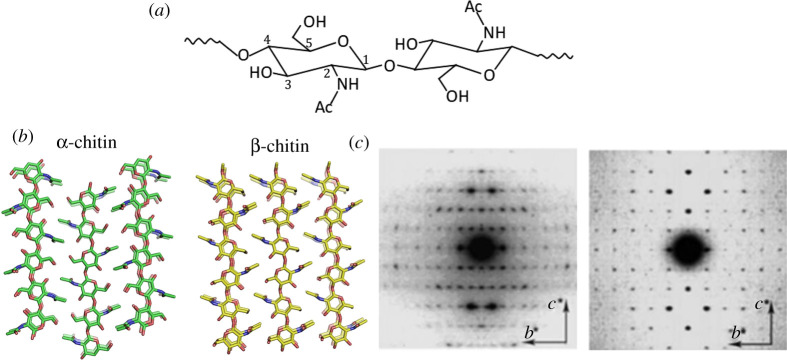
(*a*) Chemical structure of chitin. (*b*) Molecular models of the crystal structure of α-chitin and β-chitin showing the antiparallel and parallel chain directionality. Modified from [5]. (*c*) Electron diffractograms of highly crystalline chitin are taken on the grasping spine of *Sagitta*, left [6], and *Tevnia jerichonana* microcrystals, right [7]. (Online version in colour.)

In total, 8–10 billion tons of chitin are produced by living organisms on an annual basis [2], yet this vast amount of bio-resource is not available for us to use technically. This is mainly because chitin resources are generally regarded as waste matter [3]. Therefore, effective waste management strategies for chitin need to be legislated and protocols for isolation and purification at industrial scales need to be developed to use it as an advanced material globally. Nevertheless, chitin is a valuable resource, it is light weight, strong and following nature's examples we can identify diverse functionalities using material constructs at different scales. As in most biological constructs, chitin is not produced in an isolated pure polymer form but it is found as a nanocomposite material [4], figure 1, and in different crystalline conformations, figure 2. With such diverse occurrence in nature, although chitin synthesis starts as a biopolymer, it adopts many different structural conformations that lead to a number of striking optical effects such as antireflective arrays in cicada wings [8,9] or exceptional mechanical properties as in the mantis shrimp [10] and partially organized fibrils and flexible structures such as in mushrooms [11]. Organisms use the chitin's basic fibre units as structural building blocks. Through biosynthesis of chitin, these fibrils assemble in to different conformations, i.e. both as long thin microfibrils as well rod-like entities. Such differences seen in the building blocks suggest that specific forms of chitin may play an important role in creating such diverse structural roles [12].

Starting from the molecular level of interactions into supramolecular and fibrillar organization, the structural diversity and the multifunctionality of chitin is a consequence of a brilliant evolutionary optimization process. As in most fibrillar materials, chitin's internal structure is tuned and designed in a hierarchical arrangement formed through the self-assembly of their fundamental building blocks into higher-order fibre structures, which are stabilized by non-covalent interactions [13]. Figure 1 explains such hierarchical organization. The different functionalities achieved by chitin can actually arise from different interactions that take place during the non-equilibrium self-assembly processes. For example, chitin layers in insect scales cause structural coloration through the arrangement of the chitin nanofibrils in a few hundred nanometres of periodic arrays corresponding to the visible wavelengths of light [14], whereas, the mechanical strength is a consequence of load transfer between those nano- and macro-fibres [15].

Biosynthesis of chitin is a highly complex and sequential process that shows variations according to the species, nevertheless the cellular machinery of chitin synthesis is conformed in fungi and insects. Chitin synthase (UDP-*N*-acetyl-d-glucosamine:chitin 4-β-*N*-acetylglucosaminyl transferase; EC 2.4.1.16) is a membrane-integral enzyme that is found in every chitin-synthesizing organism and is the key for the biosynthetic pathway [16]. Chitin biosynthesis involves a series of enzymatic reactions that convert different sugars into a polymer of *N*-acetylglucosamine (GlcNAc) via the hexosamine pathway (HP) [17]. The HP pathway can be generalized into three distinct sets of enzymatic reactions summarized below.

**Figure 3.**
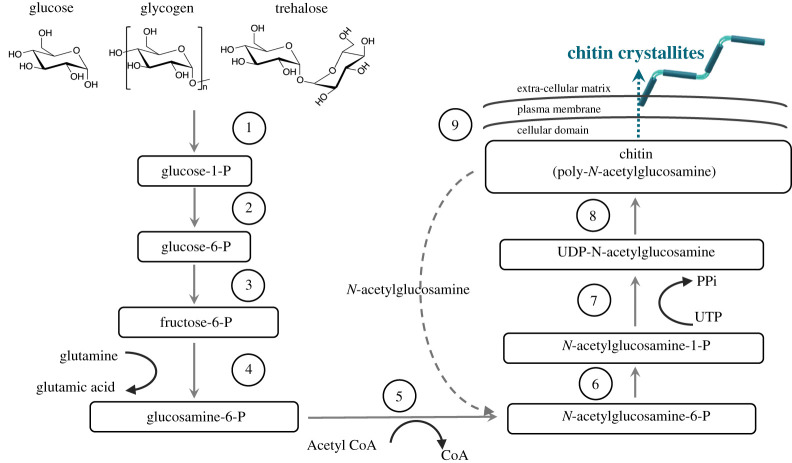
Chitin synthesis pathway in fungi and insects modified from [21,22]. (Online version in colour.)

1. The first step is the formation of the *N*-acetylglucosamine-6-phosphate sugar [18–20]; figure 1 steps 1–5. In this sequence, basic sugars such as glucose, glycogen or trehalose are converted in to glucose-6-phosphate by hexokinase and then conversion to fructose-6-phosphate takes place directed by the glucose-6-phosphate isomerase. To these intermediates amino and acetyl groups are added via glutamine fructose-6-phosphate amino transferase and glucosamine-6-phosphate *N*-acetyltransferase to form N-acetylglucosamine-6-phosphate.
2. The second sequence is the production of the activated amino sugar uridine diphosphate *N*-acetylglucosamine-(UDP-*N*-acetylglucosamine), figure 3 steps 6–7. Phosphoacetylglucosamine mutase transforms its phosphate group to convert the *N*-acetylglucosamine-6-phosphate into *N*-acetylglucosamine-1-phosphate. Subsequently, *N*-acetylglucosamine-1-phosphate is combined with uridine nucleoside triphosphate (UTP) to form UDP-*N*-acetylglucosamine [18].
3. The last sequence is the polymerization of chitin using UDP-*N*-acetylglucosamine by the action of chitin synthase, figure 3, step 8. In this final step, chitin polymeric chains are extruded along the cell membrane and released into the extra-cellular space and the polymer chains assemble to form chitin nanofibrils in the extra-cellular space, figure 3, step 9 [23].

Biosynthesis of chitin, summarized here, may look to be straightforward (from basic sugars to polymerization using a series of enzymes); however, many of the intermediate steps as well as the final stages in chitin synthesis in any organism are not very well known. Therefore, a detailed understanding of chitin biosynthesis is still lacking. In our opinion, purification of the enzymes and intermediate products during chitin synthesis for each step provides an interesting and very complex challenge not only in terms of understanding biology but also in terms of biomimicry. The natural systems work in harmonious sequential processes directed by gene expressions. Development of high-resolution direct imaging methods that are combined with chemical analysis, labelling of the saccharides and enzymes can be used to tackle such complex challenges.

In this opinion piece, we firstly discuss the basics of the crystal structure of the chitins and its chemical accessibility to develop polymeric precursors. Then, we will discuss several examples of natural photonic structures seen in nature and their different colouring mechanisms and functionality. After that we will compare various mechanical functions of chitins such as its mechanical load bearing and water repellency. Finally, we address the application space of chitin together with its most known derivate chitosan and summarize the future direction that we believe will be effective in chitin's circular economy as material.

## 2. Chitin chemistry and crystal structure

Chitin is a polysaccharide composed of 1–4 linked 2-acetamido-2-deoxy-β-d-glucopyranose, figure 2. On the evolutionary map, it is safe to say chitin is placed between cellulose and collagen. Native chitin is a semi-crystalline biopolymer that occurs in fibrous crystalline states as several polymorphs. The semi-crystalline polymer is formed through hierarchical organization; basically the polymer chains assemble into alternating amorphous and crystalline regions, figure 1*d*, which are stabilized by hydrogen bonds and van der Waals forces [13,24]. There are three allomorphic crystalline forms, designated as α, β and γ, that have been identified from X-ray diffraction studies [25,26]. The molecular and crystalline order of chitin implies the characteristics of the tissue and the physiological function of chitin. For example, the tough and stiff armour of mantis shrimps (*stomatopods*) is achieved by the α-chitin fibrils articulating themselves in to a twisted Bouligand structure of the chitin followed by mineralization and protein binding [27]. This particular arrangement gives the mantis shrimp exceptional mechanical properties to achieve flexibility, toughness and impact resistance while at the same time showcasing an exceptional evolutionary adaptation to defence and predation [28].

The α-chitin is mostly found in crustacean shells such as prawns, crabs and lobsters, as well as beetle shells and fungi cell walls [29]. The α-chitin is found to be the most stable crystalline form of chitin with polymer chains arranged in antiparallel fashion which gives rise to strong hydrogen bonding (two per unit cell), figure 2*b* [30]. The crystalline lattice structure of the α-chitin was found to be the orthorhombic space group P2_1_2_1_2_1_ with unit cell dimensions a = 4.74 Å, b = 18.86 Å and c (fibre axis) = 10.32 Å [31], figure 2*c*. β-Chitin is mostly obtained from marine diatoms [32], molluscs [33] and the peritrophic matrix in insects [34] and the polymer chains are arranged in a parallel order, figure 2*b*; the hydrogen bonds (one per unit cell) between the two chains crystallize in the monoclinic space group P21. The one chain monoclinic unit cell has dimensions a = 4.85 , b = 9.26 Å and c (fibre axis) = 10.38 Å and *γ* = 97.5°[35], figure 2*c*. The chain configuration of the β-chitin is analogous to the parallel chain polarity found in the native cellulose polymorphs. One particular point to highlight is that, while most of the naturally occurring polysaccharide crystals demonstrate parallel structure, the stable and very common polymorph of chitin, the α-chitin, demonstrates an antiparallel chain configuration and polarity. While we have more understanding established in terms of polymerization of cellulose, as we highlighted before, our general understanding on the details of chitin polymerization is limited. Still, we expect the antiparallel chain arrangement would require a very different synthetic machinery or mechanism. The polymer chain alignment also has the consequence of α-chitin being extremely aggregated through hydrogen bonding. On the other hand, β-chitin offers a more flexible and chemically ‘open structure’ in which the functional groups of chitin are oriented in a way that enables enhanced chemical activity.

By contrast, in γ-chitin, the arrangement of the chitin chains and the crystalline structure is slightly more complicated: three chitin chains, with alternating parallel and antiparallel aligned polymer chains [36]. Compared to α-chitin and β-chitin, occurrence of the γ-chitin is not very common and only in a few studies was it reported to be present in beetle cocoon fibres [37]. γ-Chitin also has monoclinic space group P21 with unit cell dimensions a = 4.7 Å, b = 10.3 Å and c (fibre axis) = 28.4 Å and *β* = 90° [38].

Chitin is chemically analogous to cellulose with acetamide groups located at the C-2 positions, figure 2*a*. Chitin crystallites are rod-shaped fibrillar units formed through supramolecular assembly of 19 molecular chains [39]. The chitin rods and the protein matrix form a natural composite together with the mineral matter. There is strong evidence that the chitin molecules actually make covalent bonds with its protein matrix so in its native form, chitin forms chitin proteoglycans [5,40]. The presence of acetyl, amino and hydroxyl groups in the polymer chain and the presence of intermolecular and intramolecular hydrogen bonds makes the chitin structure tightly bonded. Therefore, chitin does not dissolve in most regular solvents such as water, organic solvents and even mildly acidic or basic solutions. On the other hand, chitin can be easily deacetylated using concentrated caustic solutions to produce chitin which has free amino groups and is soluble. Depending on the base concentration and managing reaction kinetics, it is possible to produce partially deacetylated chitin–chitosan blends. Interestingly, we have also seen reports on organisms like fungi and invertebrates, with varying degrees of deacetylation, giving a continuum of structure between chitin (fully acetylated) and chitosan (fully deacetylated) in nature [41,42]. From a chemistry point of view in order to exploit chitin as a commercial polymer and devise chemical methods to functionalize it, it is necessary to establish efficient methods to dissolve chitin homogeneously. While some solvent combinations such as NaOH/urea or *N,N*-dimethylacetamide (DMA) with LiCl have been shown to dissolve chitin [43], we certainly think the use of corrosive and hazardous solvents should be avoided when natural products are processed. More importantly most of the mechanical and chemical transformations of chitin destroy its brilliant natural microstructure and, in our opinion, efforts should be focused on using the micro-structure of the chitin without disruption of this structure or building chitinous building blocks that can conform back into similar hierarchical organization.

## 3. Photonic chitin structures in nature

Colours play an essential role in nature in terms of communication, signalling, defence and camouflage for all living organisms [44]. While various pigments cause selective absorption of light by the delocalized electrons of molecules embedded in the material, photonic structures and structural colours are caused by different optical processes like diffraction, interference or scattering of the incident light with ‘ordered’ structures on a few hundred nanometre scale. While chitin offers the main structural constituent of the exoskeleton to the species, it is also offers a unique colour palette that is durable and responsive. Most beetles, butterflies and molluscs are therefore the source of inspiration for many research fields including optical physics, materials science and chemistry.

In nature, biological organisms tend to evolve unique structures or functions to survive and to adapt to their natural environments. Therefore, understanding the evolutionary development of the material structure can be particularly useful because it may reveal how evolutionary pressures led to the adaptation of material structure [45].

**Figure 4.**
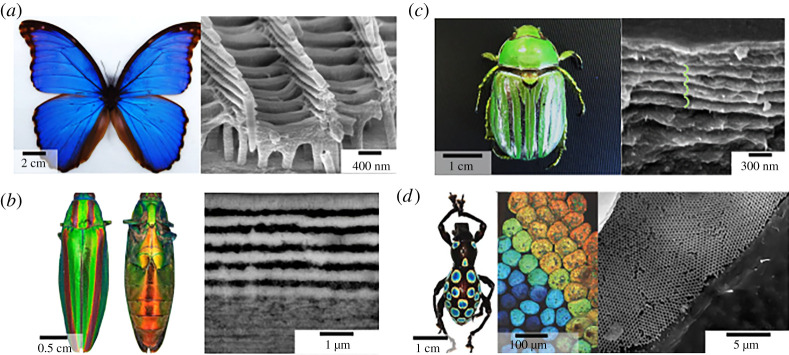
(*a*) *Morpho Peleides* butterfly image (left) and SEM image of the transverse section of wing scales, showing ridges and the ‘Christmas tree’-like structure (right). (*b*) Female Japanese jewel beetle, *Chrysochroa fulgidissima* (left). Transversal TEM sections of cuticle, showing the multi-layered structure (right)[46]. (*c*) Glorious scarab, *Chrysina gloriosa* (left) and SEM image of cuticle with multi-layered structure with helicoidal cholesteric twist. (*d*) Photograph of the snot weevil, (*Pachyrrhynchus congestus pavonius*) (left), bright-field light microscopy image (middle) and an SEM image of a single green-coloured scale (right), Modified from [47]. (Online version in colour.)

One of the most extensively studied photonic structures in terms of understanding the optics and biomimicry is the *Morpho* butterfly, with unique metallic-blue coloration [44]. The iridescent blue colour is a result of coherent scattering in the periodic arrays of scales forming a multi-layered structure. In contrast to laminar multi-layers, *Morpho* species reflect the metallic-blue colour with a low angle-dependency, which is a very unique optical phenomenon. This is caused by the presence of multi-layer surfaces that exhibit a distribution of tilts (Christmas tree-like structures) with respect to the scales' baseline, figure 4*a*. This gives a strong diffraction by a second layer of periodic ridges above the layer of highly iridescent base [48]. Organized chitin is indeed an optically anisotropic material and exhibits birefringence due to alignment of the chitin polymer fibres [49]. The butterfly cuticles are composed of chitin, with a refractive index of 1.54–1.62 [50]. To attain a multi-layered effect and the refractive index contrast, butterfly structures use the negative space filled with air. As a result, *Morpho* butterfly wings achieve a complex optical response to produce its highly reflective angle-independent brilliant blue colour.

Beetles, on the other hand, are evolved to develop complex optical signals such as iridescence, luminescence, polarized reflectance and complex photonic structures [51], which give even higher structural diversity to chitin. For example, many beetles like leaf beetles (*Gastrophysa viridula*), rosemary beetle (*Chrysolina americana*) or Japanese jewel beetle (*Chrysochroa fulgidissima*), figure 4*b*, have multilayers in the cuticle of their elytra (the covering forewings that protect the hindwings-flying wings) and body, making them highly reflective [46,52]. In scarab beetles, like glorious scarab (*Chrysina glorosia*) or demon dung beetle (*Phanaeus demon*), chitin fibres again form multilayers, but this time the layers twist in to a left-handed helicoidal structure that cause the cuticle in the elytra to selectively reflect left circularly polarized light, figure 4*c*. Generation of quasi-ordered structures can also produce structurally coloured surfaces, yet the colour response is diffused and non-iridescent such as in the case of *chrysomelids*. The way the structural colour arises is based on the scattering of light from almost identically sized light-scattering objects arranged through an evenly spaced matrix causing constructive reflectance [53]. Both the linear and helicoidally arranged beetle and butterfly shells shift colours upon interaction with water due to filling of the pores in the multi-layer with water (ref index *n* = 1.33) leading a shift in the effective refractive index of the spacer layer (*n*_average_ = 1.45 with water and *n*_average_ = 1.26 with air). Such an increase in the effective refractive index causes a red-shift in the reflectance [54].

Both for the beetles and the butterflies the one-dimensional photonic structures offer a significant advantage as layers only a few micrometres thickness are sufficient to attain a strong optical response [55]. The insect wings are multifunctional and the balance between the optical functionality, strength and flight is carefully optimized. The effectiveness of using a few micrometres thick layers in the exocuticle of the beetles is even further highlighted in white beetles (*Cyphochilus* and *Lepidiota stigma*)*.* Bright white coloration is usually achieved by multiple light scattering from a thick layer of fibres or highly refractive medium. In contrast, white beetles use 5–15 µm layers made of a very dense interconnected random fibrillar network of chitin with a relatively high filling fraction 50–60% [56]. In this case, the fibre thickness, random orientation and the filling fraction of the chitin layers is optimized to create extremely short mean free paths to achieve multiple scattering and such intense white coloration. On the other hand, the glasswing butterfly (*Greta oto*) uses chitin nanopillars with high aspect ratio arranged in a random order to achieve a completely different optical effect (transparency). For such anisotropic nanopillars, the difference between scattering incidents resulting from the longitudinal and circumferential axis cause the high transparency of the glasswing butterfly [57].

The complexity of photonic structures can get even more sophisticated: three-dimensional photonics crystals in weevils, including the single diamond and single gyroid networks made of chitin nanostructures [58].

For example in the case of the rainbow weevil (*Pachyrrhynchus congestus*), the iridescent scales and the rainbow appearance are due to a three-dimensional photonic crystal network of chitin that shows an inverse opal like structure with diamond symmetry [47], figure 4*d*. Such a configuration of chitin is particularly intriguing; experimentally the double diamond and double gyroid morphologies can be realized through a bottom-up phase transitional self-assembly of block polymers. Yet to our knowledge, there is no direct route to self-assembling single diamond and single gyroid network phases, let alone at the optically relevant mesoscale (150–300 nm) so chitin biosynthesis takes an interesting turn here and guides us through the synthesis of complex morphologies once we understand how nature tweaks an amphiphilic polymer chain to adapt such morphology.

While the beetles and butterflies are demonstrating an array of structural diversity leading the many different optical effects, many crustaceans—shrimps, crabs, lobsters, etc.—although made of similar Bouligand layers to the jewel beetles do not appear iridescent on their outer shells. There are members of the crustacean family, such as the blue crab (*Callinectes sapidus*) and the green crab (*Callinectes aestuarii*), that may be using pigments like carotenoproteins as well as the Bouligand structure like a waveguide to achieve a mixed colouring effect [59]. Such colour mixing strategies using pigmentation and structural effects are also found in butterfly wings and bird feathers to enhance the optical effects and gain further functionalities. Nevertheless, for most of the crustaceans, chitin nanofibrils are the major structural component of their exoskeletons, together with CaCO_3_, proteins and pigments, and thus the structural design of these species was driven by their mechanical performance. A detailed analysis of the dispersion of the helicoids and their chiral pitch was examined by our group and our findings show that the cholesteric pitch in different sections of crustacean shells vary between a few hundred nanometres and 2 µm. Such a difference in the pitch lengths combined with the scattering caused by the mineral phases is the possible cause for the lack of brilliant iridescent coloration on the exterior of prawn, lobster and crab outer shells. The constructive interference incidents are constantly competing with scattering and absorption caused by the structural variance and the complex chemical structure. However, once the complex proteins and mineral matter are removed the bare chitin layers provide striking optical effects. To test this theory, Nguyen & Maclachlan explored the exoskeletal structures of king crabs and converted them into hydrogel-like structures. Through the deprotenization, demineralization and layer separation stages they were able to produce flexible hydrogels with circularly polarized colour response that is somewhat similar to the jewel beetles (*Chrysina glorisia*) [60].

While the chitin-based photonic structures demonstrate these optical effects due to interference of light from approximately periodic structures, a degree of disorder can be a desired feature indeed. As mentioned before, specific optical appearances, such as angular independent colour responses or white diffuse appearances, rely on partially disordered or fully random structures [56]. From the material design point of view, there are many elements to bring together to attain such complex optical effects: the reflecting elements must be on a subwavelength scale, need to be produced on a large scale, and must be sufficiently ordered to produce the desired colour and reflectivity. In nature, most material constructs must also bear some disorder, on the subwavelength scale, in order to eliminate the directionality and sharp reflectance peaks associated with multi-layered interference. As we mentioned previously, the complexity of the biosynthesis of the chitin is one of the main challenges for a detailed understanding of at which stage they configure into specific morphologies. In order to understand evolution of colorations, mechanical properties, thermal regulation and regenerations of chitin in a larger sense, a fully multi-disciplinary approach and active collaborations are needed. Furthermore, the main driver of such structures, i.e. benefits created by optical signalling versus mechanical properties needs to be investigated in more detail.

## 4. Mechanical properties of chitin

Chitin has remarkable mechanical properties as a biopolymer demonstrating significant structural strength and flexibility for many different species optimized for specific functionalities. Many of the toughest structures that we see in nature, use chitin as an armour for protection and defence, e.g. insect cuticles, crustacean shells and mollusc nacre [61,62]. Such toughness is a result of the hierarchical construct of high-strength chitin in a protein matrix reinforced by the CaCO_3_ minerals, such as in the case of the carapace of the mantis shrimp [63]. For the mantis shrimp the stiffness and hardness of the exoskeleton is optimized via the mineral matter to chitin ratio as well as chitin's binding with its surrounding protein network [64]. Covalent bonding of the proteins with the chitin fibrils directs the conformation and at the same time the biomineralization process hardens and stiffens the whole construct. Many arthropods regularly change their exoskeletons and produce new armour on a regular basis [65]. On the other hand, butterfly wings do not have such intertwined hard/soft composite to maintain their flight function, so the wings are only made of light matter: a chitin matrix with proteins. The wings still exhibit excellent strength and durability with respect to their low density. The study of these complex assemblies and understanding of the interplay between micro- and macro-structural property relationships is the key to understanding bionics and developing high-strength novel materials for future technologies.

We have summarized some key studies that show the mechanical performance of chitin from different species in table 1. Comparing Young's modulus and tensile strength between arthropods and beetles reveals the essence of the inorganic matrix and bonding between the chitin and the protein matrix which causes a decrease of the modulus for beetle elytra [69,70]. For many of the species, alignment of the chitin and the final hierarchical framework defines the ultimate strength, which can change due to the chitin content and crystallinity. This has been confirmed: the variation of *Pachynoda sinuata* modulus indicates orientation of the chitin fibres and also determines the modulus. The multi-layer structure of insect cuticles might also help to improve the strength and toughness. Skykes *et al.* [76] studied the elytra in *Macraspis lucida* to investigate how cracks propagate in a multi-layered structure. Interestingly, the multilayers of the *M. lucida* are arranged in 90° alignment of the chitin fibres in each layer. They have shown that the fibre-lamina structure and interface in cuticles can frustrate crack growth, protecting the elytra from a catastrophic break, which provides a key survival tool. One thing to bear in mind is that chitin is an amphiphilic polymer and sensitive to hydration content; therefore, the mechanical properties of chitin strongly depend on the hydration state; both Young's modulus and tensile strength properties show about 20–30% fluctuation between the dry and wet states [71] Again, understanding the mechanism of formation of such sophisticated mechanical structure conduits is extremely significant in developing bioinspired and bioinformed materials.

**Table 1.** Mechanical properties of native chitin from different sources, nanofibrillated chitin and chitin nanocrystals.

material type/chitin source	hydration state	Young's Modulus (GPa)	tensile strength (MPa)	crystalline structure and crystallinity %	ref
fibre-chitin from snow crab (*C. opilio*)	dry	59.3	−	α-chitin	[66]
mantis shrimp saddle chitin and protein bound	dry	240		α-chitin	[64]
mantis shrimp saddle chitin only	dry	80		α-chitin	[64]
fibre–cellulose	dry	86	−	Cellulose I	[67]
fibre–kevlar	−	85	−	−	[68]
beetle elytra–*Potosia brevitarsis*	dry	5.45	−	−	[69]
beetle elytra–*Allomyrina dichotoma*	dry	4.34	130	−	[69]
beetle elytra–*Pachynoda sinuata*	dry	0.09–1.52	36–69	−	[70]
regenerated chitin (RC) films/nanopapers–crabs	wet	0.33	20	−	[71]
RC films/nanopapers–crabs	dry	1.095	36	−	[71]
RC films/nanopapers–squid pan(Illex argentines)		6.7	277	89% β-chitin	[72]
RC films/nanopapers–α-chitin from crabs	dry	9	156.5	α-chitin	[73]
RC films/nanopapers–prawn shells	dry	7.3	89.4	α-chitin	[74]
RC films/nanopapers–mushroom (*Agaricus bisporus*)	dry	6.9	204.4	−	[75]

Nature has optimized the chitin and the inorganic/organic nanocomposite in the most efficient way. This means chitin is embedded in to a complex chemical matrix. Therefore, during the extraction and purification processes, whether chemical, microbial or enzymatic, it has significant influence on the mechanical properties of the final chitin-based products (films and fibres). Furthermore, the extraction process also subjects the chitin resources into harsh mechanical treatments such as intensive crushing and grinding for large-scale production of chitin. All of these processes disrupt the hierarchical structure of the exoskeleton and cause a drastic loss in mechanical properties [3]. Again in table 1, we include the composite matrix of the regenerated chitin films/nanopapers that have much smaller Young's modulus, which vary between 6 and 9 GPa [71–75]. Wu *et al.* [72] and Montroni *et al.* [77] explored the excessive chemical treatment and indeed reported 15% decrease in their mechanical performance. If the fibrillar structure of the chitin is used in its native form using mechanical fibrillation techniques, chitin fibrils can offer a number of excellent properties such as an axial fibril modulus of around 60 GPa [66]. One of the key factors for conservation of mechanical properties is keeping the hydrogen bonding between the chitin acetyl groups during such treatments. Molecular dynamics studies by Cui *et al.* [78] support the same opinion that the degree of acetylation can determine mechanical properties. As building blocks, chitin whiskers, which can be prepared from chitins, also show great mechanical properties. The longitudinal modulus and transverse modulus of individual chitin whiskers were 150 and 15 GPa, respectively [79,80], which makes chitin whiskers a potential filler for reinforcing in polymer composites. Generally, mechanical strength is very important for durability.

The strength of chitin is mainly from hydrogen bonding formed between an acetyl amine group and a hydroxyl group. Meanwhile, an investigation [81] into α-chitin from American lobster (*H. americanus*) shows the textures have a preferred arrangement to provide the highest protection. To our knowledge, differences between the mechanical strength between the α-chitin and β-chitin and interplay between the crystallinity, hydrogen bonding and amorphous chain design in chitin is still not well established. In our opinion, developing models around crystallinity, their mechanical strength and interplay with different functionalities would be an important area to understand the structural diversity of chitin.

**Figure 5.**
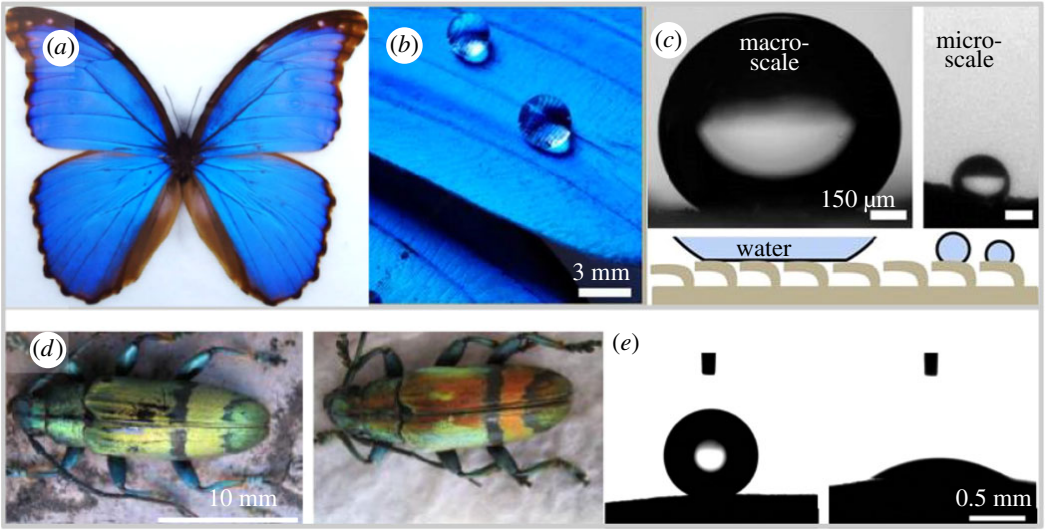
(*a*) *Morpho Peleides* butterfly image. (*b*) Interaction of water droplets on the superhydrophobic scales. (*c*) Two different water repelling mechanisms based in the scale on the left the butterfly scales trap an air layer collectively, resulting in a high contact angle of 160° ± 10° for millimetric droplets and on the right an individual scale is shown with micrometre scale droplet showing the nonlinear surface properties. Adapted with permission from [83]. (*d*) Longhorn beetle *Mesisternus isabellae* in the dry/left and wet/right states. (*e*) Contact angle measurements for the black/left band and coloured/right region of the elytra [84]. (Online version in colour.)

In addition, chitin, as an amphiphilic polymer, can show superhydrophobic properties based on the micropatterning and surface engineering at the nanoscale [82]. For example, many butterflies' wings are superhydrophobic, figure 5*a*–*c*. This wonderful property might arise from the arrangement of numerous nano-tips on nano-stripes, which form quadrate scales that covers the butterflies’ wings periodically [85] creating a barrier for water and contact solely exists with the tips of the structural elements fitting the Cassie–Baxter theory [86]. Depending on the wing position with respect to the droplet falling angle this effect can change; when the wings are tilted up the Wenzel state can apply with water being in intimate contact with all of the rough surfaces [87]. Inspired by the microstructural hydrophobicity, in 2014 Duan *et al.* [88] synthesized a superhydrophobic chitin sponge by coating methyltrichlorosilane (MTCS). In another approach, chitosan micro-particles were spray coated on a silicon wafer to attain superhydrophobicity with a water contact angle of 155 ± 1° [89]. Such studies are extremely interesting as they offer a new approach using micro- and nanostructures for selective fluidic interactions; furthermore, both approaches offer development of sustainable, low-cost compostable functionalities. While butterflies are using their wings and chitin to attain various flight, colour response and self-cleaning, beetles, on the other hand, take a different approach on water management on their elytra. Most beetles have superhydrophobic performance that is generated by a wax layer [90], which is thin and chemically hydrophobic on a relatively smooth surface, which eliminates the need for micro-patterning. In other beetles like the longhorn beetles (*Tmesisternus isabellae*) however the scales of the elytra show bands of hydrophobic and hydrophilic regions, figure 5*d*–*e*, which shows water infiltration and absorption are as important as self-cleaning for the beetles' resource management metabolically [84]. Chitin, as a high-strength polymer material, draws attention from many fields and will continue developing unexpected applications. In the next part, we will discuss some of the fantastic applications of chitin.

## 5. Chitin-based materials for future advanced technologies

Nature showcases a plethora of ideas for us to use chitin as an advanced material with its hierarchical structure, multifunctionality, brilliant light management and extraordinary mechanical properties. Yet chitins are not primarily used in the fields of optical material development, or as a smart structural material. Some of the obstacles in commercialization and application of chitin in these areas arise from challenges with the isolation of chitin from its native structure; the removal of proteins requires treating the exoskeleton or the shells with a basic solution such as dilute NaOH or KOH and a demineralization process involving the use of a variety of acids like HCl, HNO_3_ or H_2_SO_4_. A more critical problem though is the mixing of different resources of chitin for mass production and the mechanical treatments used prior to the removal of the biochemical matrix, which disrupts the hierarchical structure of the chitin and consecutively affects the mechanical and optical properties.

**Figure 6.**
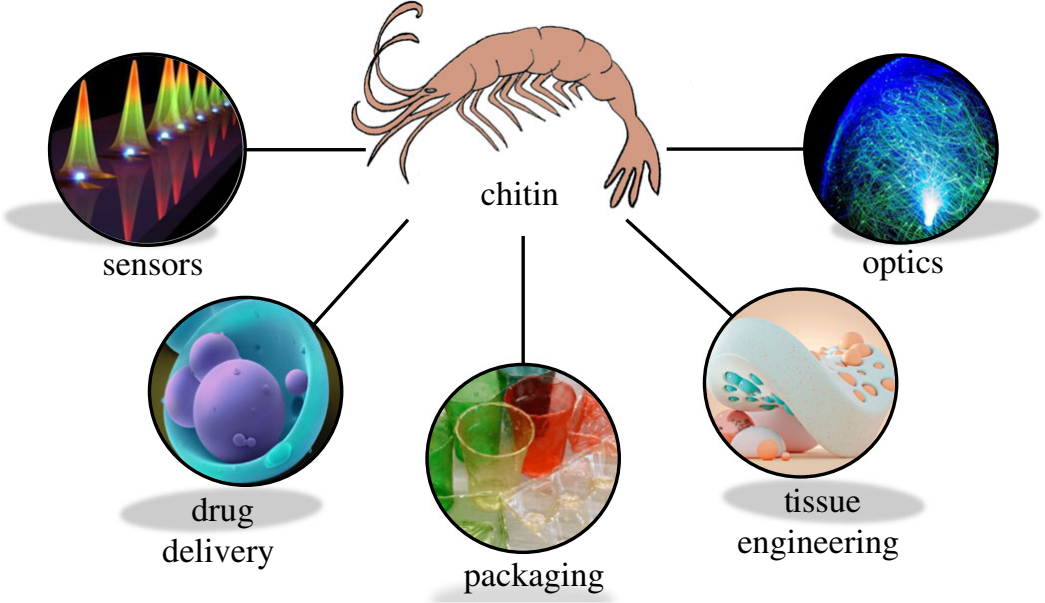
Schematic representation of the application areas for developing chitin-based material systems, using chitin's intrinsic qualities for development of sustainable solutions. Figures are reproduced with permission from the following sources. Sensors image is reproduced with permission from the authors [91]. Drug delivery image ‘Microparticle drug delivery’ by Annie Cavanagh is licensed under CC-BY-NC 4.0. Available from: https://wellcomecollection.org/works/df6vfbzr. Packaging image reproduced with permission from Wyss Institute at Harvard University. Available from: https://news.harvard.edu/gazette/story/2014/05/promising-solution-to-plastic-pollution/. Tissue engineering image provided by Roman Bratschi. Available from: romanbratschi.com. Optics image is modified from ‘Fiber optical lamp globe’ by Groman123 and is licensed under CC BY-SA 2.0. 2015 Available from: https://www.flickr.com/photos/85203634@N05/15653529233. (Online version in colour.)

Nevertheless, as a functional biopolymer, chitin has excellent potential to be used in various forms such as films, fibres, cross-linked hydrogels and functional components in composites; we have summarized some key future directions for chitin-based technologies in figure 6. The chitinous building blocks include polymer preforms, nanocrystals and nanofibres. Such structural and processing versatility and its highly desired intrinsic properties like low density, high tensile strength, biocompatibility, biodegradability, and nontoxicity [92] makes chitin an ideal choice for a wide spectrum of applications in varying areas, such as biomedical, packaging and optical materials. As a biopolymer, one of the most promising areas for using chitin is as a biomedical material, in tissue scaffolds, drug delivery systems and wound healing patches [93–99]. Chitosan is also used to produce nanofibres for possible scaffold applications where electrospun chitosan nanofibres promote adhesion of mouse osteoblasts and potentially can be used to treat bone defects and fractures [95]. In these applications, chitin is usually used as a polymer in a dissolved form using electrospinning, through cross-linking or decellularization of the native tissues themselves. There is also an emerging trend to manufacture three-dimensional printed chitosan scaffolds in the field which can be used for soft tissue repair [100] and chronic dermal wounds [101]. Chitin is intrinsically antimicrobial [102] and accelerates wound healing through facilitating macrophage migration and fibroblast proliferation, which promotes granulation and vascularization [103,104].

Chitin's inherent mechanical properties as well as its transparency make chitin and its derivatives a suitable candidate to replace single-use plastics. Our studies show that chitin breaks down in seawater in four to eight weeks and such natural decomposition activity is highly desirable and addresses a very problematic area of the packaging industry, i.e. plastics in the ocean. Chitin being antifungal and antimicrobial is also highly preferred to manage food preservation performance and tune the gas and water barrier properties further. We strongly believe that biopolymer-based packaging solutions hold a prominent future. Especially, for the case of chitin, since the raw material (shrimp shells versus crab shells) is sourced from waste material streams. This addresses two main problems which are problematic for the seafood industry: (i) a waste product is valorized as a sustainable material and (ii) the shellfish waste is removed from the waste management streams. This also highlights the importance of understanding the circularity and end life assessments in offering new materials for future technologies. In order to create an efficient market for chitin as a biopolymer that is competitive to fossil-based synthetic polymers, chitin extraction methods from different sources need to be optimized. We believe further research efforts need to focus on formulating bulk processing methods with low carbon and chemical footprints while producing a high purity chitin from natural sources without disrupting its brilliant hierarchical structure.

Another promising application area for chitin-based films is to develop green optics and sensors. As we have described in the photonic properties of chitin, nature uses chitin to produce iridescent wings with dynamic colour response, a highly scattering nano-fibrillar medium as well as transparent shells. Such functionality offers many solutions to several industries. For example, for packaging materials and display matrices, transparency is highly desired. This can be achieved by mimicking the structural arrangement of naturally transparent materials such as in the example of krill and prawn shells [105]. The transparency of these shells is caused by the interplay between the nanofibre size, porosity and filling factor against the visible light wavelengths to modulate scattering effects. When the size of the nanofibres is much smaller than the range of visible light in terms of diameter, transparency can be achieved through optimization of the mean pathway of the light scattering. Overall, these phenomena enable the production of transparent chitin films [106,107] which can further enable development of flexible displays [105,108]. An important aspect of reported studies with chitin reinforcement and chitin-based films is that manufactured films were still able to maintain their flexibility [108–111]. Precise control of flexibility, transparency and electrical response of chitin-based composites is also very promising for chitin films in flexible OLED substrates [109]. In the field of sensors, a reported novel application is chitin-based contact lenses combined with graphene field-effect transistor to track blood sugar content in the rabbit tear fluids [110]. Also by using chitin's mechanical strength, a scratch resistant screen cover window based on chitin has been reported [110].

While nature shows an array of materials with striking optical properties, the biomimicry of such diverse structures is not usually achieved by chitin as a building block and the chitin photonics field has not been much developed. We find it presents an interesting contrast to cellulose photonics as only a few cellulosic species such as *Pollia condensata* [112], *Margaritaria nobilis* [113] and *Lunaria annua* [114] are found to display natural photonic structures. Yet the studies using cellulose nanocrystals as a building block for photonic applications is vast. In contrast, reports on making use of chitin as a colloidal building block for photonics [115] or using it as a template [14] are limited. Production of a two-dimensional cholesteric film using chitin as a building block that is optically active in the IR region [115] has recently been reported by Vignolini *et al*. Using chitin nanocrystals as building blocks for photonics requires a thorough understanding of building block properties, i.e. size distribution, suspension stability and their interaction in different ionic strengths. Our own studies in this field indicate the possibility of fabricating photonic structures with optical activity in the visible region.

## 6. Conclusion and outlook

In this opinion piece, we have attempted to showcase the structural diversity of chitin in nature and presented an overview of its structure, complexity and its functionality in nature. In each section, we addressed the questions that we felt would drive biomimetic and bioinformative research fields to further improve our understanding of chitin synthesis and evolutionary design of these intricate infrastructures in nature and their translation to real materials that can be used in different industries.

Chitin and its derivative chitosan are two of the most abundant functional polysaccharides on earth, demonstrating great diversity in their structure and chemistry. The way nature uses chitin as a structural fibre with many different functionalities will inspire many different areas of research for many years to come. From the biosynthesis point of view, sequential processes for polymerization and crystallization are still big questions. In particular, the puzzle of the formation of completely different bioarchitectures from similar synthesis processes such as the Christmas tree-like nanostructures versus gyroid formations in butterflies has not been solved. Understanding the *in vivo* development of such structures from gene expressions, enzymatic activities as well as the chemical matrix employed in different stages of the biosynthesis will allow us to shift the material design paradigms. Certainly, developing methods that allows us to follow the structure progress in chitins would be a significant scientific contribution, yet the complexity of the biology requires a collaborative and multi-disciplinary research effort.

The crystalline structure of chitin derives many different properties from mechanical strength to birefringence. From our point of view, it is very interesting to debate whether the optical effects or the mechanical properties have driven the evolutionary pathways to give structural diversity. One way to test this would be a correlative study of high-resolution imaging of the structural development *in vivo* with precise biochemical analysis. Efforts needs to be focused on chemical and fluorescent tagging of the proteins that are instrumental in the dynamic non-equilibrium growth processes as well as non-destructive imaging systems to follow the growth. Following nature's footsteps in using supramolecular assembly as well as the secondary forces to achieve mechanical balance between stiffness and flexibility, we can translate these soft matter correlations into solid-state materials and composites.

We believe chitin has a great potential in replacing fossil-based polymers for packaging applications as well as developing smart materials that can respond to their environment (sensing and signalling). For such application areas, chitin needs to be graded (based on its source) and produced with a high degree of purity. One key area we should bear in mind is that while nature has evolved to create these structures, isolation of chitin breaks down this finely tuned structure and the functionalities that arise from such sophisticated structures are lost in using chitin as a bulk produced biopolymer. This brings another exciting research challenge for us to discover ways to isolate chitin from its native environment with minimal chemical intervention. For future advanced technologies using chitin will ultimately drive many innovations and alternatives using biomimicry in materials science.

## Data accessibility

This article does not contain any additional data.
